# Retracted: Differential Control of Growth, Apoptotic Activity, and Gene Expression in Human Breast Cancer Cells by Extracts Derived from Medicinal Herbs *Zingiber officinale*

**DOI:** 10.1155/2023/9838617

**Published:** 2023-01-08

**Authors:** BioMed Research International

BioMed Research International has retracted the article titled “Differential Control of Growth, Apoptotic Activity, and Gene Expression in Human Breast Cancer Cells by Extracts Derived from Medicinal Herbs *Zingiber officinale*” [1]. As initially raised on PubPeer [2], the article was found to contain concerns in Figures 2, 4 and 5. Specifically:

- In Figure 2 the right-hand side of 2(a) 0.05 mg/mL appears to be rotated 180 degrees and duplicated in the right-hand side of Figure 2(b) 0.1 mg/mL. A white line is also visible in Figure 2(b)0.1 mg/mL
- In Figure 2(b) a section of the image representing 0 mg/mL appears to be rotated 180 degrees and replicated in the image representing 0.025 mg/mL
- In Figure 4(b) there appears to be undeclared splice sites in the gels for Bcl-2 and Bax. In addition, both HPRT bands appear identical

The authors were contacted for clarification, but they were unresponsive. The article is therefore retracted from the journal with the agreement of the Editorial Board due to concerns regarding the reliability of the data.
